# Gene Duplication Analysis Reveals No Ancient Whole Genome Duplication but Extensive Small-Scale Duplications during Genome Evolution and Adaptation of *Schistosoma mansoni*

**DOI:** 10.3389/fcimb.2017.00412

**Published:** 2017-09-21

**Authors:** Shuai Wang, Xing-quan Zhu, Xuepeng Cai

**Affiliations:** State Key Laboratory of Veterinary Etiological Biology, Key Laboratory of Veterinary Parasitology of Gansu Province, Lanzhou Veterinary Research Institute, Chinese Academy of Agricultural Sciences Lanzhou, China

**Keywords:** gene duplication, genome, *Schistosoma mansoni*, evolution, adaptation

## Abstract

Gene duplication (GD), thought to facilitate evolutionary innovation and adaptation, has been studied in many phylogenetic lineages. However, it remains poorly investigated in trematodes, a medically important parasite group that has been evolutionarily specialized during long-term host-parasite interaction. In this study, we conducted a genome-wide study of GD modes and contributions in *Schistosoma mansoni*, a pathogen causing human schistosomiasis. We combined several lines of evidence provided by duplicate age distributions, genomic sequence similarity, depth-of-coverage and gene synteny to identify the dominant drivers that contribute to the origins of new genes in this parasite. The gene divergences following duplication events (gene structure, expression and function retention) were also analyzed. Our results reveal that the genome lacks whole genome duplication (WGD) in a long evolutionary time and has few large segmental duplications, but is extensively shaped by the continuous small-scale gene duplications (SSGDs) (i.e., dispersed, tandem and proximal GDs) that may be derived from (retro-) transposition and unequal crossing over. Additionally, our study shows that the genes generated by tandem duplications have the smallest divergence during the evolution. Finally, we demonstrate that SSGDs, especially the tandem duplications, greatly contribute to the expansions of some preferentially retained pathogenesis-associated gene families that are associated with the parasite's survival during infection. This study is the first to systematically summarize the landscape of GDs in trematodes and provides new insights of adaptations to parasitism linked to GD events for these parasites.

## Introduction

Gene duplication (GD) is a very common phenomenon in all eukaryotic organisms and has been generally viewed as an important force in species evolution (Ohno, 1970; Zhang, 2003). It occurs by several modes, mainly including unequal crossing over (giving rise to tandem or proximal GD), (retro-) transposition (giving rise to dispersed GD), and whole genome/chromosomal duplication (WGD) (Zhang, 2003; Kaessmann, 2010; Magadum et al., 2013). Generally, the most obvious contribution of GD is providing new genetic material for functional and structural evolution. It facilitates increases in gene functional diversities (neo-functionalization or sub-functionalization) and contributes to gene dosage effects, by both of which GD can be proposed to be adaptive when organisms are confronted with stress (Zhang, 2003; Innan and Kondrashov, 2010; Chang and Duda, 2012). Especially, lineage specific duplications, which are derived from duplication events along specific lineages after splits, can underlie some of the key phenotypic characteristics that distinguish species an provide adaptations to specific evolutionary niches (Fortna et al., 2004; Meyer and Van de Peer, 2005; Hanada et al., 2008). In addition, nearly identical genomic regions derived from duplication events provide hotspots for chromosomal rearrangements that permit rapid changes to occur during evolution (Bailey et al., 2002; Kim et al., 2008). Consequently, a genome can be extensively shaped by GD, and its plasticity in adapting to changing environments can be significantly increased during this process (Zhang, 2003).

The roles of different GD modes in genome evolution have been investigated in many organisms (Kondrashov et al., 2002; Cheung et al., 2003; Maere et al., 2005; Freeling, 2009; Chang and Duda, 2012; Lu et al., 2012; Wang et al., 2013; Rensing, 2014; Vanneste et al., 2014; Cardoso-Moreira et al., 2016). In particular, WGDs or large-scale duplication events have been extensively studied in increasingly complex organisms (e.g., vertebrates or plants; Panopoulou and Poustka, 2005; Vanneste et al., 2014), due to their attributed importance in evolutionary transitions and adaptive radiations of species. Other GD modes, defined as small scale gene duplication (SSGDs) in this study, are also found to contribute to the origin of a substantial portion of genes in many species (Freeling, 2009; Innan and Kondrashov, 2010; Chang and Duda, 2012; Lu et al., 2012; Wang et al., 2013; Cardoso-Moreira et al., 2016). In addition, some duplicated genes have been demonstrated to play central roles in adaptations to some specific niches, such as, Dca gene that is involved in adaptation to lower temperature in *Drosophila* (Arboleda-Bustos and Segarra, 2011), and major histocompatibility complex (Burri et al., 2010) and immunoglobulin gene families (Guldner et al., 2004) that are likely linked to host-pathogen interactions. For an evolutionarily specialized group, parasites have undergone long-term adaptations and evolved a lot of specialized mechanisms to interact with their hosts during life cycles (Tsai et al., 2013; Jackson, 2015; Zarowiecki and Berriman, 2015; Wang et al., 2016). Therefore, it will be particularly important to investigate the nature and extent of GD in genomes of parasites to better understand the associations between GD events and evolutionary adaptations. In fact, the duplications or expansions of some specific genes have been found to play essential roles in antigenic variation, invasion or host-parasite interactions during infection (Foth et al., 2014; Hull and Dlamini, 2014; Cwiklinski et al., 2015; Zarowiecki and Berriman, 2015; Lorenzi et al., 2016). However, to date, genome-wide knowledge on fundamental biological processes or origins of GD is still poorly investigated in most parasite groups, especially in trematodes which are among the most neglected tropical pathogens with great medical and economic importance.

Here, we present a genome-wide analysis of GDs in *Schistosoma mansoni* (a blood fluke from the class trematode) to summarize the predominant GD patterns and evolutionary contributions. This parasite is one of the major infectious agents responsible for the chronic debilitating disease schistosomiasis in human. Its genome and associated annotations have undergone iterative improvements (Berriman et al., 2009), which currently represents the best refined genome in the class, and thus creates a valuable opportunity to investigate GDs in trematodes. In this study, a combination of computational methods were used to identify the GD modes and estimate the subsequent evolutionary consequences in the genome. The results provide the first blueprint of GDs in the genomes of blood flukes. Our findings firmly highlight that the genome have undergone no WGD or large-scale GD events in a relatively long-term evolution and has been extensively shaped by ubiquitous SSGDs from unequal crossing over and (retro-) transposition. In addition, our study indicates that SSGDs, especially the tandem duplications, contribute greatly to the expansion of several pathogenesis-associated gene families that are preferentially retained in this parasite. These results provide new insights into the genome evolution pattern of flukes and give a better understanding of the adaptations to their environments.

## Materials and methods

### Data preparation

The complete genome sequence (ASM23792v2), gene model and gene expression data (version 2016-05-WormBase) of *S. mansoni* were obtained from WormBase (http://parasite.wormbase.org/index.html). If alternative transcripts were available in the gene model, only the one with the longest CDS was kept. The genes with premature termination codons (*n* = 18; most of which are mitochondrial genes) in their coding sequences or flagged as pseudogenes (*n* = 15; which are supported without any evidence to provide a function) in the annotations were excluded. This resulted in a dataset of 10752 sequences in total for further analyses. Illumina paired-end raw reads (ERR266713) were retrieved from NCBI (ftp.ncbi.nlm.nih.gov).

### Construction of empirical *Ks* age distribution

The potential WGDs or SSGDs were inferred from duplicate age distributions by the use of *Ks* (the number of synonymous substitutions per synonymous site), based on a refined method as described by (Vanneste et al., 2013). An all-to-all sequence similarity search was performed using BLASTP (*E*-value ≤ 1e^−10^). Gene families were subsequently built through Markov Clustering (Enright et al., 2002) using the mclblastline pipeline (micans.org/mcl) with an inflation value 2.0. For each paralogous gene pair, a protein alignment was constructed using MAFFT (v7.147b) (Katoh and Standley, 2013) and was then converted into the DNA sequence alignment. The gene pairs were retained only if the two sequences were alignable over a minimum gap-stripped length of 100 amino acids with an identity score of at least 30%. *Ks*-values were estimated using the CODEML program (mode = 0 and runmode = −2) implemented in the PAML package (Yang, 2007), each of which was repeated five times to avoid suboptimal estimates of the global maximum likelihood. Large families were subdivided into subfamilies if *Ks* between genes exceeded a value of 5.0. An average linkage clustering approach was used to correct the redundancy of *Ks* values (a gene family of n members produces n [n–1]/2 pair-wise *Ks* estimates for n-1 retained duplication events), as described in Vanneste et al. (2013). Briefly, for each family, a tentative phylogenetic tree was constructed by average linkage hierarchical clustering, using *Ks* as a distance measure. Each split in the resulting tree can represent a single gene duplication event and can be weighted by a single corrected *Ks*.

### Segmental duplication detections in the genome

To further identify segmental duplications (SDs) in the genome sequence, all chromosomes were directly aligned with one another using the Nucmer algorithm (–mumreference -o -p) implemented in the MUMmer package (Kurtz et al., 2004). The hard-masked version of the genome (ASM23792v2) was used to exclude high-copy repeats (e.g., LTR and LINE elements) in the analysis. In this study, recent large segmental duplications were defined to be ≥10 kb in size and ≥90% in sequence identity (Bailey et al., 2004).

Nearly identical duplicated sequences might have been erroneously collapsed within the genome. To assess its potential effect on the estimation of origins of duplicated genes in this study, we further used an assembly-independent method by examining their whole-genome shotgun read coverages. Adapters and low-quality reads (ERR266713) were removed or trimmed by FastX-toolkits (http://hannonlab.cshl.edu/fastx_toolkit/). The high-quality clean reads were aligned to the genome reference by Bowtie2 (-I 100 -X 600) (Langmead and Salzberg, 2012). The potential PCR or optical duplicates were removed by Picard (http://broadinstitute.github.io/picard/) from the produced BAM file, followed by a second-round filtering by Samtools (Li et al., 2009) with the pair-end information fixed. The individual read coverage was compared to the mean read coverage over the entire genome, which was approximately 37×. Before the analysis, we assessed the distribution of the coverage of each position in the assembly and found it a nearly normal distribution (Supplementary Figure 1), indicative of a nearly uniform distribution of the mapped reads over the genome. Therefore, the coverages of unique regions should have a Poisson distribution with a mean coverage of 37. For the truly duplicated sequences, the depth-of-coverage shows a statistically significant increase due to recruitment of paralogous reads. We employed a sliding window strategy with a class of 5 kb windows and a sliding of 1 kb window (Bailey et al., 2004) to count the read depth over 5 kb windows. Initial calls were selected if the observed average coverage of a continuous sequence is at least twice the normal coverage and the read depths of most of its sites (≥70%) were high than that cutoff. Neighboring regions would be merged over the gap if the interval is smaller than 2 kb.

### Identification of gene duplication modes

Intra-species collinearity blocks were also detected by MCScanX algorithm (Wang et al., 2012) with default settings (5 genes required to call a collinear block) based on the previous all-to-all BLASTP result (*E*-value ≤ 1e^−10^). The origins of paralogous gene copies (i.e., duplication categories of whole genome/segmental, tandem, proximal or dispersed duplications) were determined using the duplicate_gene_classifier program implemented in the MCSanX package. The dispersed gene duplications may occur via DNA or RNA-based mechanisms, and the latter mechanism, often known as retro-transposition, generates intron-less retrocopies (Zhang, 2003). To explore the evolution of dispersed duplications that may directly originate from a retro-transposed mechanism, the potential poly-A tracts within 3 kb after the stop codons in the intron-free dispersed members were detected by a sliding window analysis (window size = 30 nt, shifted by 1 nt). A poly-A signal was reported when the percentage of adenosine sites were larger than 90% within a window. In addition, the signature of intron loss was also used to differentiate a duplicate derived from retrosposition (Zhang et al., 2011). For each intron-less gene, we chose the gene with the most identical amino acids for all alignments (see Construction of empirical Ks age distribution section) as the best hit, reducing the data set to the one best hit per intron-less gene. We then kept only gene pairs in which the single exon gene's best hit had multiple exons (potential parental gene). Finally, an intron-less gene was regarded as a putative retrogene, if its alignment was long enough to cover exon–exon junctions within its intron-containing parental gene.

### Gene divergence, functional and positive selection analysis

To understand how structural divergence between duplicated genes changes over evolutionary time, gene structural divergence between duplicated genes identified by MCScanX was measured by differences in coding-region lengths, average intron lengths and number of introns in the gene models. The transcriptomic information available in Protasio et al. (2012) was used for transcription expression divergence between duplicated genes. The expression levels of genes were evaluated by fragments per kilobase of exon per million fragments mapped (FPKM) values. Wilcoxon Signed-Rank test, implemented in R (using the option paired = YES), and Spearman's correlation coefficient (*R*-value) were used to compare and measure the differences. Gene ontology (GO) terms for each gene were assigned by the InterProScan program (Jones et al., 2014) and GO enrichment for each gene set was calculated by BLAST2GO (Conesa et al., 2005), using the whole gene data set as reference. The over-representation and underrepresentation of certain GO terms were analyzed based on Fisher's exact test (False Discovery Rate, FDR ≤ 0.05).

The maximum likelihood method implemented in PAML (Codeml; mode = 0; runmode = −2) was used to determine the synonymous (*Ks*) and non-synonymous (*Ka*) substitution rates for each paralogous pair. Subsequently, the ratio of *Ka*/*Ks* was used to identify genes under positive (*Ka*/*Ks* > 1) vs. negative (*Ka*/*Ks* < 1) selection pressure. Gene pairs showing *Ks* values ≥ 2 or ≤ 0.01 were excluded, as such high or low *Ks* values may result in inaccurate *Ka*/*Ks* estimates. The gene pairs showing abnormally high *Ka*/*Ks* values (>10) were discarded. In addition, the site model implemented in Codeml was used to evaluate selection pressure for each paralogous group with three or more members (see the section Methods), using the strategy described in the respective article (Emes and Yang, 2008). For the paralogous families for site model analysis, large families were subdivided into subfamilies if *Ks* between genes exceeded a value of 2.0 which indicates saturation of substitutions. The phylogenetic tree for each group was reconstructed by Neighbor-joining method. Two pairs of models were used: M1 (neutral) vs. M2 (selection); and M7 (beta) vs. M8 (beta+ω). For each pair of nested models the log likelihood values were compared using the likelihood ratio test with 2 degrees of freedom. Codons were identified as undergoing adaptive evolution if both tests were significant (*p*-value ≤ 0.05).

## Results

### Gene duplication distributions of the *S. mansoni* genome

**Figure 1:**
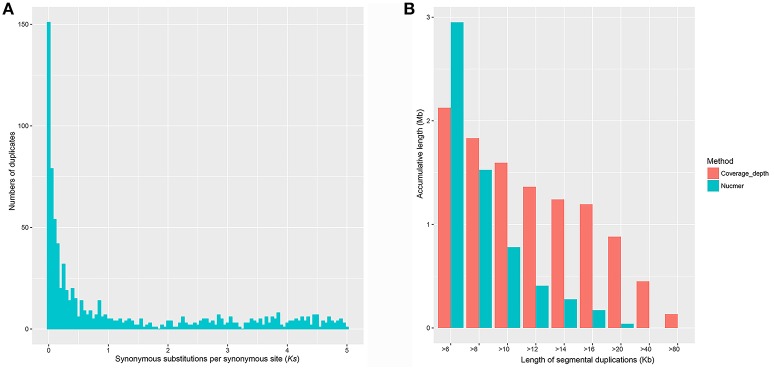
Gene duplications in the *S. mansoni* genome. **(A)** Empirical *Ks*-based age distributions for *S. mansoni*. The *Ks* distribution of paralogous genes in the *S. mansoni* genome is typically L-shaped, which agrees well with the notions that most duplications are young. **(B)** Segmental duplications identified by Nucmer and coverage-depth methods. Very limited numbers of large segmental duplications were identified.

Using a method based on *Ks* age distribution, we estimated the GDs in the genome of this parasite. Overall, 1013 paralogous groups were constructed after a series of filtering steps, involving 4569 genes (42.49%) as multi-copy genes. About half of them (500/1013) contain two members. Variation of the cutoff values of the blast searches (e.g., *E*-value ≤ 1e^−10^ and *E*-value ≤ 1e^−5^) and other filtering steps (data not shown) in reasonable scales could not significantly change the obtained clustering groups. Each corrected *Ks* can (each split in the resulting tree) represent a relative age of a duplication event (see Methods section). As shown in Figure 1A, the *Ks* distribution of paralogous genes in the *S. mansoni* genome is typically L-shaped. The result reveals that most of the paralogous genes are newly duplicated and have experienced rapid losses. Whole-genome duplications (WGDs) or large-scale gene duplications (LSGDs) are frequently inferred from peaks against a small-scale duplication background. However, no significant peak of genes was observed in the *Ks*-distribution for the *S. mansoni* genome, suggesting the small-scale gene duplications (SSGDs) are probably dominant in a long-term period (*Ks* ≤ 5).

### Identification of segmental duplications

**Figure 2:**
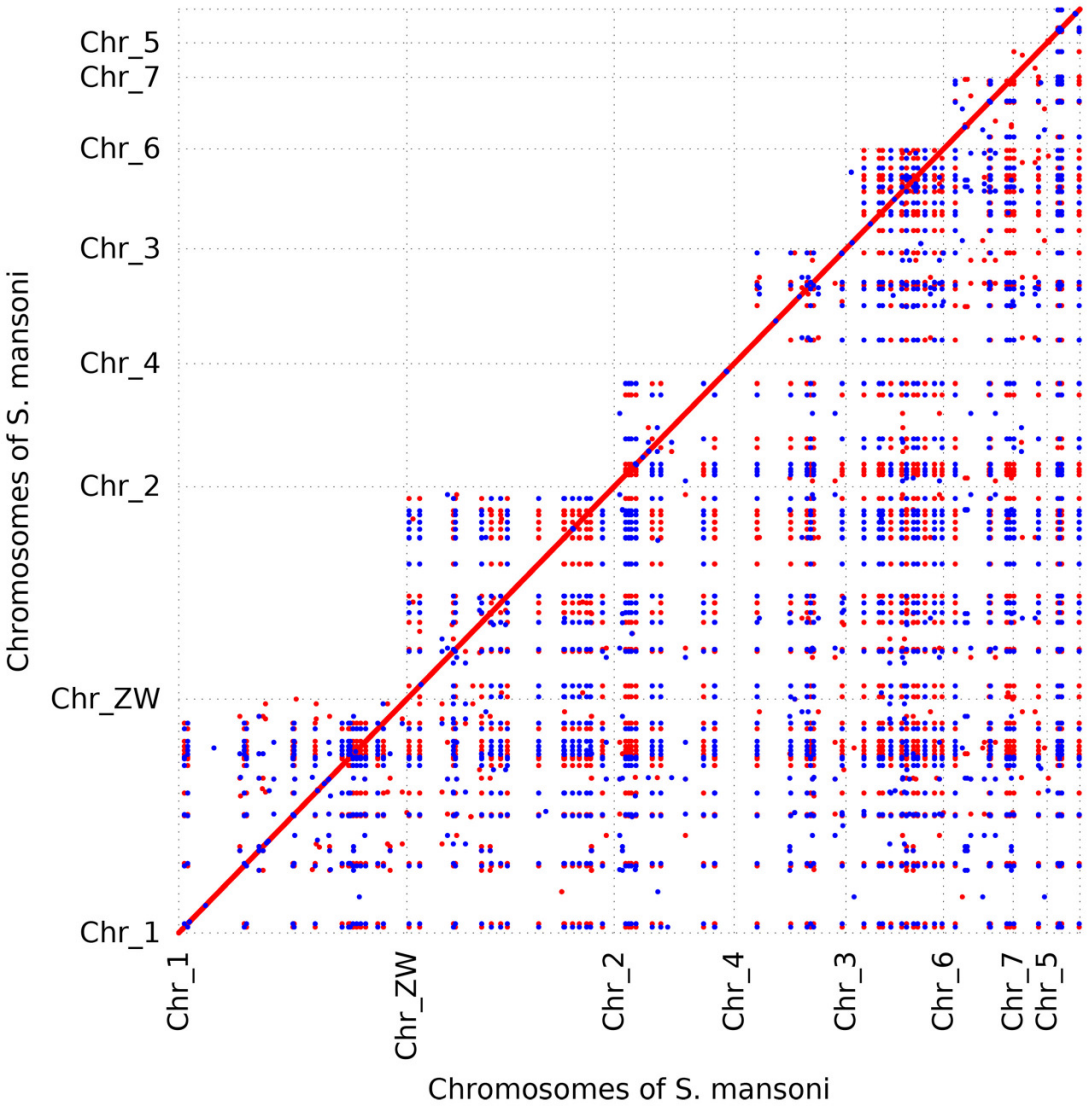
Dot plot of recent segmental duplication blocks in *S. mansoni* genome. The blocks were determined by Nucmer. Orientation is diagrammed with different colors. Only the blocks ≥5 kb are shown. A high proportion of these sequences are retrotransposons that are widespread in the genome. The tandem dots suggest that the genome has experienced substantial small tandem duplications. Some genomic regions seem to be duplicated in all the chromosomes.

To further determine whether large segmental duplications have occurred in the genome, we used Nucmer to construct a self-alignment of the genome sequences. Only 59 SDs (≥10 kb and ≥90% identity) could be identified by Nucmer with an average length of 12.29 kb and an average identity of 94%, involving 780 kb genomic sequences and 14 genes fully contained within them. The maximum SD region is ~20 kb in length (Figure 1B). The number of SD region would increase to 447 (4.15%) for the analysis if the cutoffs were reduced to be ≥1 kb and ≥90% identity, accounting for about 10.02% (~37.43 Mb) in the assembly, indicative of substantial small scale sequence duplications. Typically, these small duplicated genomic regions appear in tandem in the chromosomes of *S. mansoni*, with chromosome ZW having the highest tandem duplications (Figure 2). Interestingly, some specific homologous genomic regions are highly duplicated within a chromosome and across the whole genome. A high proportion of these sequences could be identified as retrotransposons.

In the coverage depth-based method, appropriately 97% of the high-quality reads were aligned to the reference genome, revealing that almost all the genomic regions have been successfully assembled into the assembly, at least merged into collapsed regions. We finally identified 71 regions with high read depth (≥10 kb; ~177 × average coverage fold) as potential merged SDs with an average length of 22 kb and a maximum length of 133 kb (within the contig Smp.SC_0037), approximately representing 0.4% sequence (1.59 Mb) and 45 genes in the current assembly. Duplications could be found in almost all the chromosomes, with chromosome 1 having the highest, and chromosome 6 having the least duplicated content. Substantial amounts of the duplicated content were also found in the unmapped chromosome sequence (*n* = 56), suggesting that the correct chromosomal assignment of these segments remains an assembly challenge. In this study, we roughly estimated the merged sequences to represent about 7.6 Mb of true sequence by comparing the expected read depth with the observed read depth in SD regions. As inferred from the observation, the effect on gene content estimation from the collapsed regions is limited, indicative of the high quality of the assembly. As shown in Figure 1B, the genome assembly likely contains a limited number of erroneously merged large SDs, but a series of collapsed small scale duplications.

### Gene duplication modes in the genome

**Figure 3:**
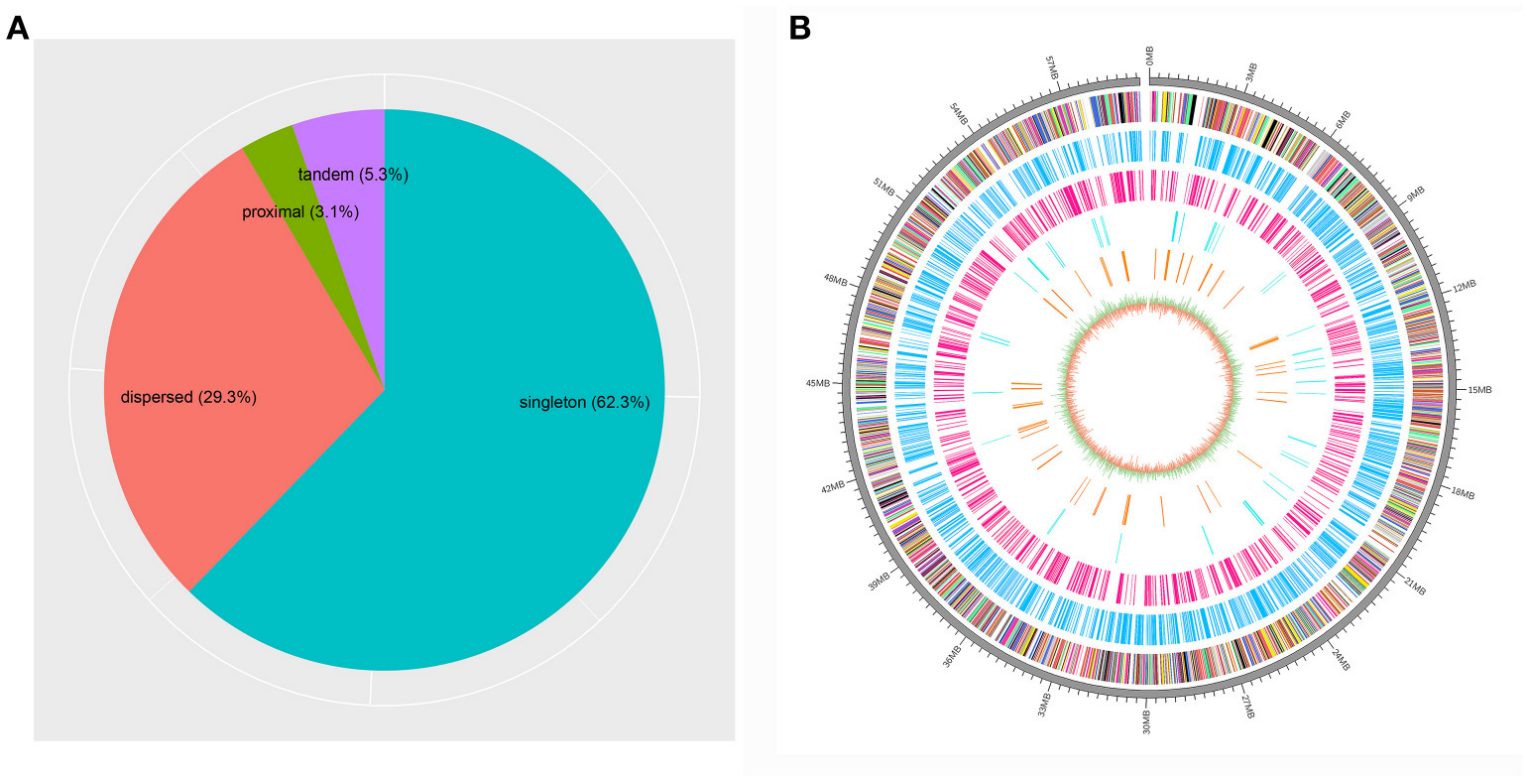
Proportions of different gene duplication modes among the genome and locations for the duplicated genes on the sex chromosome. **(A)** The gene duplication modes determined by MCSCANX are shown. **(B)** The locations of the genes in the scaffold Smp.Chr_ZW are shown for all the genes (multicolored) as well as singleton (deep sky blue), dispersed (deep pink), proximal (cyan2) and tandem duplications (dark orange). The innermost circle represents the GC content and GC skew of the genomic regions.

**Figure 4:**
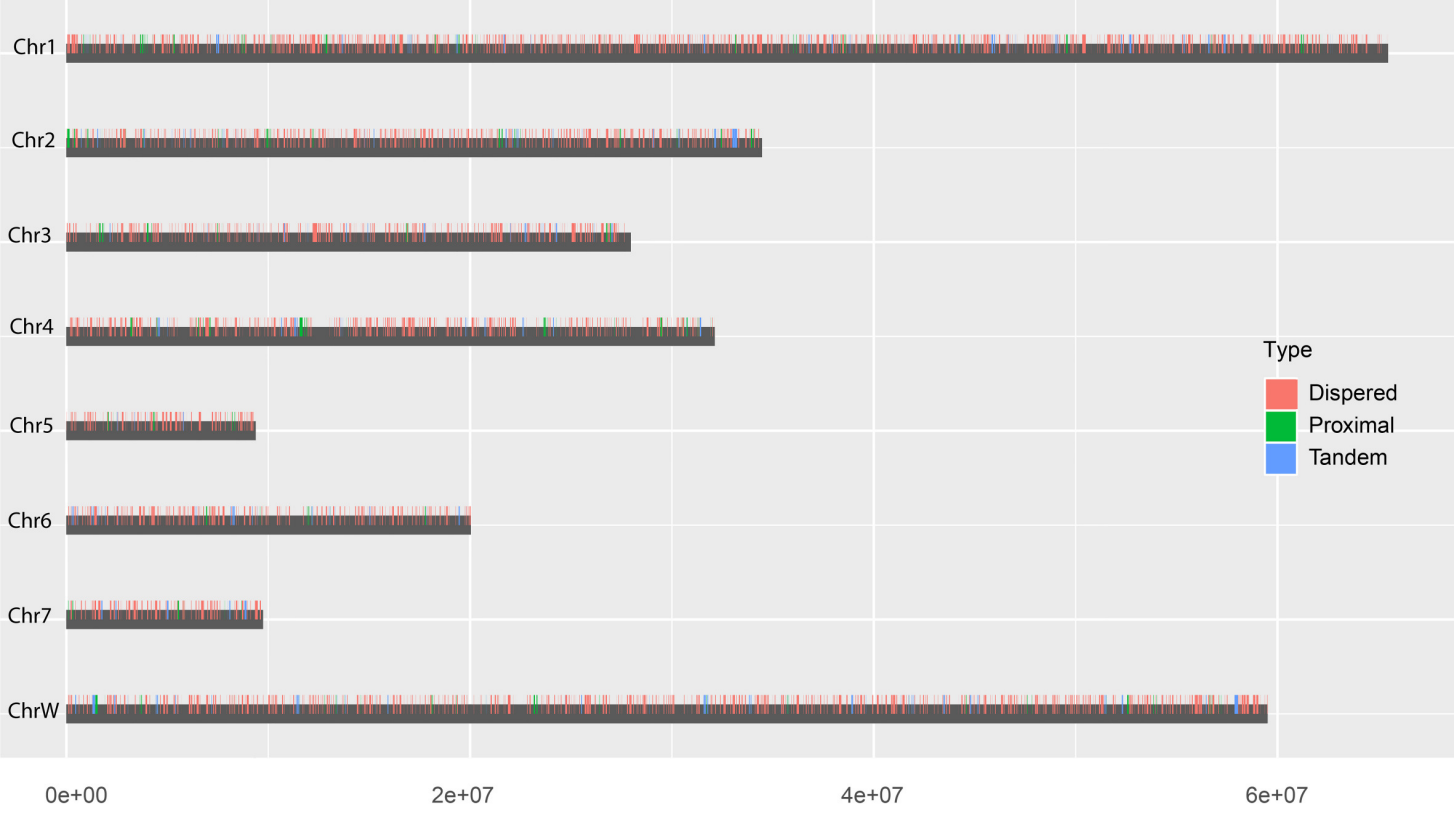
Chromosomal locations of each duplication type. The vertical bars indicate the locations of dispersed (red), proximal (green) and tandem (blue)duplications in chromosomes.

**Figure 5:**
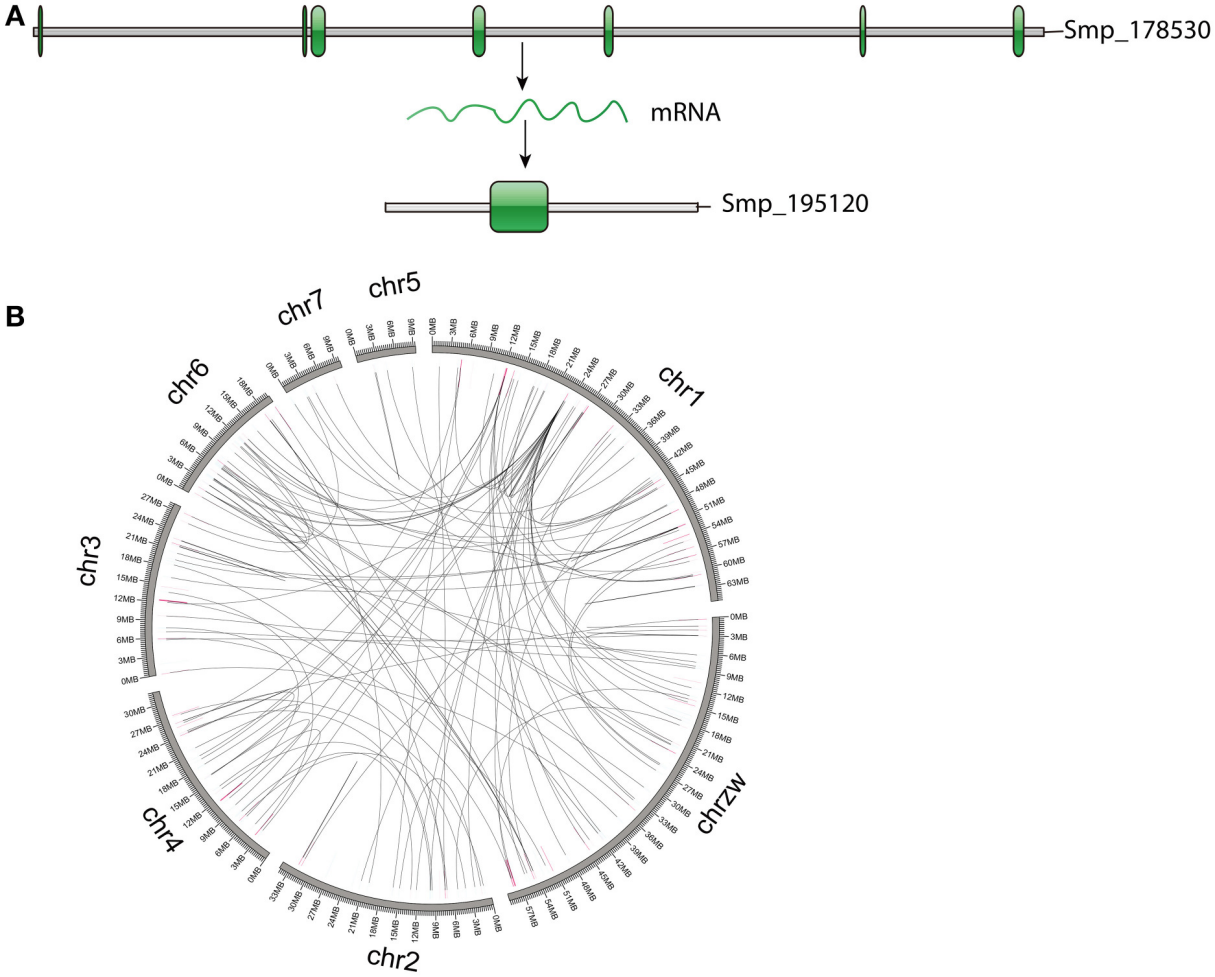
Genes generated by retroposition mode. **(A)** An example of genes identified as retrogenes. The parental gene (Smp_178530) has multiple exons, while the retrogene (Smp_195120) only has one exon, implying an origin of retroposition. **(B)** Locations of retrogenes and their parental genes. The highlights represent locations of retrogenes (blue) and parental genes (pink).

Based on the estimation by the coverage-depth method that the collapsed regions only involved limited numbers of genes, we employed the MSCANX program to explore the origins of paralogous gene copies in the assembly. Consistent with the result inferred from the *Ks* age-distribution method, no segmental duplications that contain at least 5 genes in collinearity blocks were identified in this analysis. The gene duplication events in the genome are mostly derived from SSGDs, predominated by dispersed duplications (*n* = 3462) followed by tandem (*n* = 632) and proximal duplications (*n* = 370) in the current assembly (see Figure 3A). The dispersed duplications are uniformly distributed among all the chromosomes, but the proximal or tandem duplications seem to be more likely to occur at some genomic regions (Figure 3B). As shown in Figure 4 and Supplementary Figure 2, the dispersed mode is predominant across all chromosomes and tandem duplication mode is less frequent as opposed to proximal. Within each chromosome, the density of each duplication type also varies among different genomic regions. For all the intron-less genes (*n* = 1886) in the genome, 235 genes were identified as putative retrogenes that may be generated by retroposition (Supplementary Table 1). As shown in Figure 5, these retroposition processes can be intra or inter-chromosomal. Interestingly, some genes are more likely to be parental genes that have given rise to a few retrogenes. For instance, the gene Smp_160980.1 was predicted to be as the parent gene for 42 retrogenes, while its exact function is still unknown. Of the genes generated from dispersed duplication, 223 members have a single exon, of which 175 members were predicted as putative retrogenes. Most of them (87.44%) are expressed in at least one life stage, indicating that they are evolutionarily retained as functional genes. As expected from the retroposition process, the presence of poly-A tracts may be detected if the duplication occurs very recently. However, only three genes (Smp_195040, Smp_029620, and Smp_039600) still contain such a signal.

### Gene evolution following duplications

**Figure 6:**
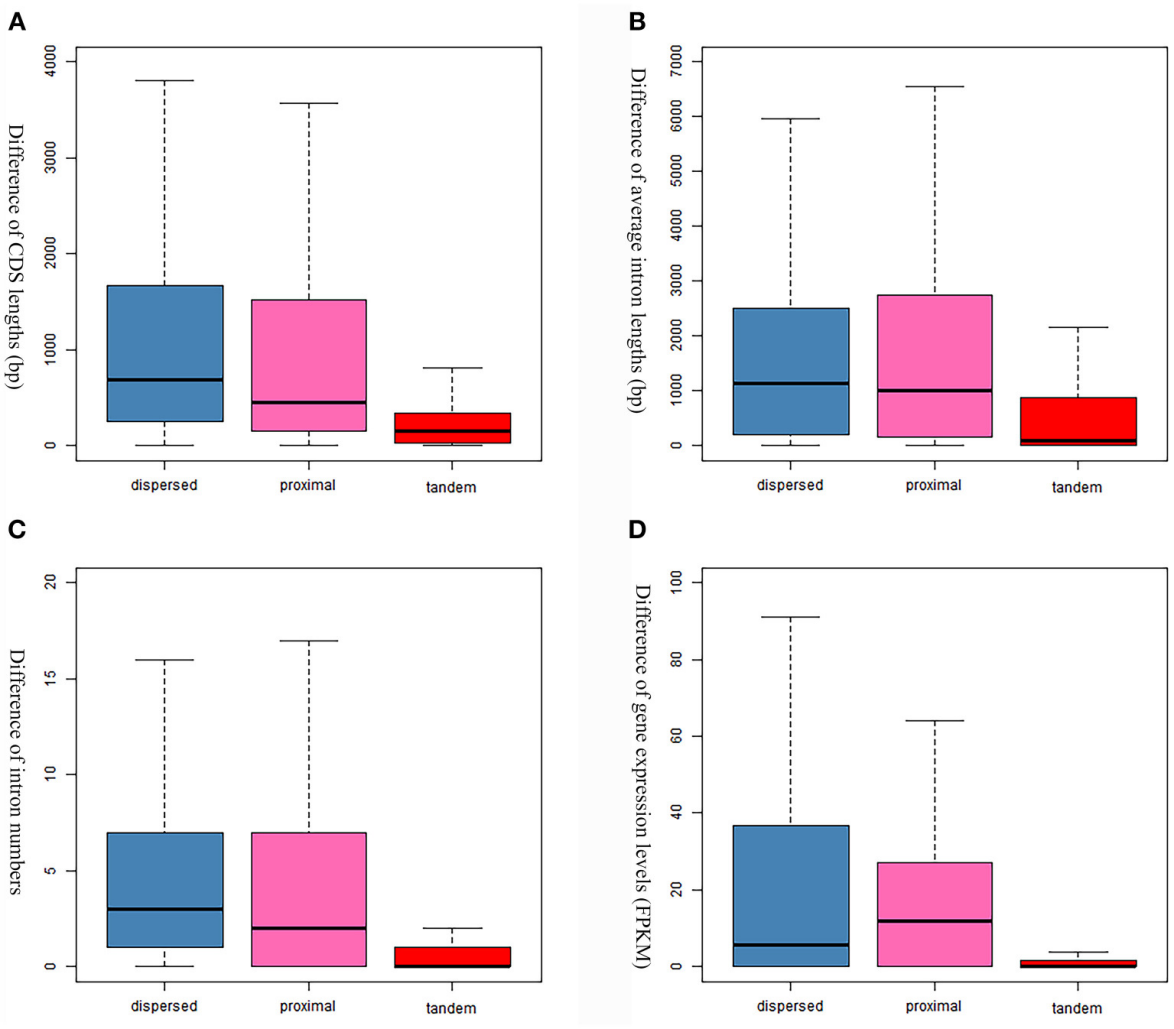
Comparison of gene evolution following gene duplications. **(A)** Comparison of coding sequence lengths. **(B)** Comparison of average intron lengths. **(C)** Comparison of intron numbers. **(D)** Comparison of expression levels that are evaluated by FPKM values.

Evolutionary consequences (gene structural divergence, expression divergence, and functional retentions) following gene duplication were investigated in this study. The dispersed GDs show the highest gene structural changes (i.e., CDS length, intron number and average intron length) in the *S. mansoni* genome, while the tandem GDs show the lowest (Figure 6). Similar tendency was also observed in the comparison of expression levels (Figure 6). Particularly, the expression levels between the tandem gene pairs are highly similar, implying correlations in expressions among them (*R* = 0.6839273, *P*-value = 2.2e^−16^).

**Figure 7:**
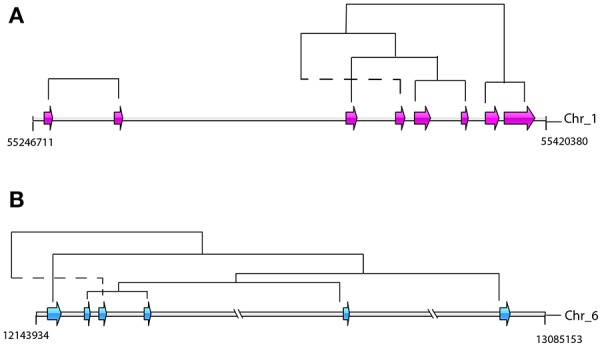
Genomic organizations and origins of venom allergen proteins-coding genes. The phylogenetic relationships were determined by reconstructions of phylogenetic tree based on the whole gene family. Dotted lines indicate potential rearrangement of duplicated genes. **(A)** Tandem duplications of tegument-allergen-like proteins in chromosome 1. The pink arrows indicate the order and location of the genes (Smp_086470, Smp_086480, Smp_086530, Smp_169190, Smp_169200, Smp_195090, Smp_072620, and Smp_072630). **(B)** The tandem and proximal duplications of venom allergen-like proteins in chromosome 6. The blue arrows indicate the order and location of the genes (Smp_176180, Smp_176160, Smp_120670, Smp_154260, Smp_154290, and Smp_070250).

Retention of the duplicated genes after duplication is unlikely to occur randomly in *S. mansoni*. For the dispersed duplicated genes, the nucleosome component and biological processes of protein phosphorylation and potassium ion transport are among the most specific enriched GO terms (FDR ≤ 0.05) (Supplementary Table 2). For the proximal duplications, the membrane components and the processes of protein phosphorylation and protein glycosylation are highly enriched (Supplementary Table 3). However, the tandemly duplicated genes have been preferentially retained for other GO categories (Supplementary Table 4). For instance, the molecular processes of G-protein coupled receptor signaling pathway, proteolysis and iron ion transport are significantly enriched in the tandem duplications (Supplementary Table 4). Interestingly, some tegumental venom allergen proteins (Chalmers and Hoffmann, 2012), such as venom allergen proteins (*n* = 12/17), venom allergen-like proteins (*n* = 8/18), and tegument-allergen-like proteins (10/13), as well as several pathogenesis-related antigens, such as tegumental antigen (Cardoso et al., 2008) (*n* = 1/3) and major egg antigens (Cass et al., 2007) (*n* = 9/12), have undergone substantial tandem duplications over evolution (Supplementary Table 5). These genes mainly appear in clusters within the chromosomes. They are probably generated by unequal crossing over between chromosomes, some of which seem to have been subject to subsequent chromosomal rearrangements (Figure 7). In addition, some invasion-related proteases (Dvorak et al., 2008), for example cercarial elastase (S01 family), cathepsin B peptidase (C01 family), invadolysin (M08 family), and hemoglobinase (C13 family), as well as some unassigned proteases from A01 and M13 families, have also been highly expanded by tandem duplication mode. This evidence revealed that duplicated genes, in particular of the tandemly duplicated genes, are non-randomly retained and may evolve vital functions for the fluke.

Overall, 322 gene-pairs (1.71%) with *Ka*/*Ks* value >1 were identified by the pairwise comparison (Supplementary Figure 3) and 18 paralogous groups (10.78%) showed positive selection signal determined by site model analysis. Most of these genes encode proteins with unknown functions (Supplementary Tables 6, 7). For candidate genes that may have potential to interact with host, egg protein CP391S-coding genes and the venom allergen proteins-coding genes are subject to positive selection detected by pair-wise model and site model, respectively.

## Discussion

Several processes, such as tandem duplications, segmental duplications or even entire genome duplications, can greatly shape genomes by leading to an increased number of genes and diversifying genome structures (Ohno, 1967, 1970; Maere et al., 2005; Magadum et al., 2013). In this study, we explored the presence and organization of such processes in the *S. mansoni* genome and provided the initial estimation of their potential contributions to its genome evolution and parasitism. This is the first systematic analysis of gene duplication in trematode species. Our results revealed several interesting features of gene duplications-both biological and practical that have not been characterized previously.

WGDs (or paleopolyploidizations) have been reported in most evolutionary lineages and are viewed as a road toward evolutionary success (Ohno, 1970; Vanneste et al., 2014). Both angiosperm and vertebrate ancestors have undergone at least two separate WGDs (Ohno, 1993; Panopoulou and Poustka, 2005; Vanneste et al., 2014). In many other kingdoms, such as, the ciliate *Paramecium tetraurelia* (Aury et al., 2006), and the hemiascomycete *Saccharomyces cerevisiae* (Wolfe and Shields, 1997), WGDs have also been documented. However, the present analysis based on the L-shaped *Ks* age distribution revealed that paleopolyploidies are probably absent in the fluke lineage in a relatively long evolutionary time (Ks ≤ 5) (Figure 1). It is consistent with the case of the investigation of paralogous genes in tapeworms that no sudden peak was observed in *Ks* age distributions (Wang et al., 2016). This estimation is further confirmed by the result from the collinearity block analysis that no genomic region with more than 5 genes was successfully called by MCScanX algorithm, indicative of absence of large segmental duplications. Because of limitations of the two methods that are highly depended on the quality of the gene prediction, we pursued two other independent methods to make further assessments. The MUMmer analysis indicated that the assembly contain very limited number of large scale duplications and more importantly, these genomic regions involved few genes. However, the duplications can be underestimated due to potential collapses of the nearly identical regions in the genome assembly. In our analysis, there were indeed some of the duplicated loci involving in potential sequence assembly errors and requiring further mapping and sequencing to achieve accuracy. But our results from the coverage depth method point out that this effect is probably limited on the gene content estimation because most of the erroneously assembled regions only contained few genes. Based on all the evidence, we believe that WGD has probably not contributed substantially to the origin of duplicated genes in this species.

Another noteworthy observation in our analysis is that the *S. mansoni* genome have experienced extensive and continuous SSGDs over evolutionary time, which is a dominant force driving the genome evolution and contributing to a substantial portion of the genes. This finding can be supported by all the methods employed in this study, including *Ks*-age, Nucmer and coverage-depth based estimations. Interestingly, this is also the case for the genomes of other protostomes, such as *Drosophila* and Caenorhabditis, and ancestral deuterostome lineages, such as *Branchiostoma* (Amphioxus) (Meyer and Van de Peer, 2005). These species also tend to have smaller gene families, often two per gene family, or only even single copies of genes, while the genomes of mammals typically have more genes, often three or four per gene family (Meyer and Van de Peer, 2005). The SSGDs can occur frequently in a genome usually by unequal crossing over or (retro-) transposition (Hurles, 2004; Kaessmann, 2010). In the *S. mansoni* genome, we found the dispersed duplicated genes are dominant across all the modes (Figure 3B). The (retro-) transposition processes, which relocate duplicated genes to new chromosomal positions via either DNA or RNA-based mechanisms, usually contribute to the widespread existence of dispersed duplicates. Intron-loss as a typical molecular feature of retroposition (Zhang, 2003) can be observed in innumerous putative retro-transposed derived genes. Using this feature, we identified 235 intron-less genes, implying that these genes may derive from retroposition, most of which were classified into dispersed duplications. Meanwhile, this result implies that the retroposition processes also contribute to the origins of genes in other duplication modes. In addition, the results also reveal an interesting phenomenon that some genes are more like to be parental gene during retroposition processes, which is observed in this species for the first time. Although the functions of these genes are unknown, they have undoubtedly greatly shaped the genome and are worth of further profound investigations. However, few Poly (A)-tract signals were found in the gene structure of the dispersed duplicated genes in our analysis. This may be partially attributed to the fact that the original signal from the (retro)-transposed mRNA can be rapidly erased during evolution. In our analysis, the tandem or proximal GDs which are typically derived from unequal crossing over have also frequently occurred in the genome (Figure 3B). In particular, the *S. mansoni* genome has been extensively shaped by recent tandem duplications which are located at all the chromosomes. Some genomic regions were highly enriched with these sequences, suggesting that hotspots for tandem duplications may exist in the genome. This is common if tandem duplications result from homologous recombination between paralogous sequences (Hurles, 2004). The location and density of tandem duplications further support their ununiform distribution along each chromosome (Figure 4 and Supplementary Figure 2). Interestingly, the genes derived from tandem or proximal duplications diverge less in gene structure and expression level than the dispersed duplicated genes. A likely explanation is that most observed tandem/proximal duplicates are relatively younger, of which the linear arrangements are still better retained. Alternatively, the observed structural divergence between duplicated genes may be greatly affected by the mechanisms of gene duplication in the *S. mansoni* genome.

The result of enrichment analysis revealed that retention of these duplicated genes did not occur randomly. Following duplication, genes belonging to some functional categories have been preferentially retained in the genome. These genes with some specific functions (e.g., protein phosphorylation, protein glycosylation and ion transport), especially the membrane components could contribute to more complex interactions and gene networks without doubt and plausibly facilitated the survival during evolution of the parasite (Prince and Pickett, 2002). Among these genes, some members have been viewed as keys to aid invasion, initiate feeding, facilitate adaptations and mediate modulation of the host immune response. Included amongst these proteins are the tegumental venom allergens (Chalmers and Hoffmann, 2012), pathogenesis-related antigens (Cass et al., 2007; Cardoso et al., 2008) and invasion-related proteases (Dvorak et al., 2008), most of which are generated from tandem duplication. For instance, the venom allergen proteins, and tegument-allergen-like proteins can interact heavily with host immune system and play central roles in the *S. mansoni* invasion and immune evasion (Cass et al., 2007; Cardoso et al., 2008; Dvorak et al., 2008; Chalmers and Hoffmann, 2012). Given the important roles of these genes in the host-parasite interaction, the remarkable contributions of tandem duplication mode to survival of this parasite can be supposed. Positive selection signal was also detected from this family, further implying its potential role in the adaption to environments (Clark, 1994). These observations indicate tandem duplication mode plays a critical role in the adaptations to parasitism for *S. mansoni* and this process is substantially underpinned by preferential retentions of duplicated genes from SSGDs.

This study provides a landscape of the recently duplicated gene content of the *S. mansoni* genome. Our results revealed that WGD is absent in this species. Extensive and continuous SSGDs have contributed greatly to its genome evolution. In addition, the results suggest the critical roles of gene duplication in adaptations to parasitism of this parasite. These findings of the genome evolution pattern will be useful in highlighting the most dynamic content of any genome assembly and the new insights will give a better understanding of the adaptations to environment challenge faced by the fluke. Furthermore, as this parasite leads to a global health problem and an incalculable drain on the economic development of endemic countries, the findings in this study may provide novel insights for the development of new intervention tools against human schistosomiasis.

## Author contributions

SW, XZ, and XC designed the research. SW conducted the analysis. SW, XZ, and XC wrote and revised the manuscript.

### Conflict of interest statement

The authors declare that the research was conducted in the absence of any commercial or financial relationships that could be construed as a potential conflict of interest.

## Supplementary material

Supplementary Figure 1 Distribution of the coverage of each position in the assembly. The coverage of each position the *S. mansoni* genome assembly was calculated by Samtools, based on the filtered clean reads (see section Materials and Methods).

Supplementary Figure 2 Proportions of each duplication type along the chromosomes. The number of each duplication type (or singleton genes) within every continuous 1 Mb region along each chromosome was counted by a window-sliding analysis. The axis represents the locations at the end of each window (Mb).

Supplementary Figure 3 *Ka* and *Ks* values for paralogous pairs. The dots with different colors represent genes under positive selection (blue) and under negative selection (red).

Supplementary Table 1 Functional annotations of putative retrogenes and their parental genes.

Supplementary Table 2 The significantly enriched GO terms of dispersedly duplicated genes (FDR < 0.05).

Supplementary Table 3 The significantly enriched GO terms of proximally duplicated genes (FDR < 0.05).

Supplementary Table 4 The significantly enriched GO terms of tandemly duplicated genes (FDR < 0.05).

Supplementary Table 5 Annotations of genes generated from tandem duplications.

Supplementary Table 6 Positively selected genes identified by pair-wise comparison.

Supplementary Table 7 Positively selected gene families identified by site model analysis.
