# Supplementary figures and images for: Gene Duplication Analysis Reveals No Ancient Whole Genome Duplication but Extensive Small-Scale Duplications during Genome Evolution and Adaptation of Schistosoma mansoni
Source: Front Cell Infect Microbiol. 2017 Sep 21;7:412. doi: 10.3389/fcimb.2017.00412 (PMC5613093; doi:10.3389/fcimb.2017.00412)

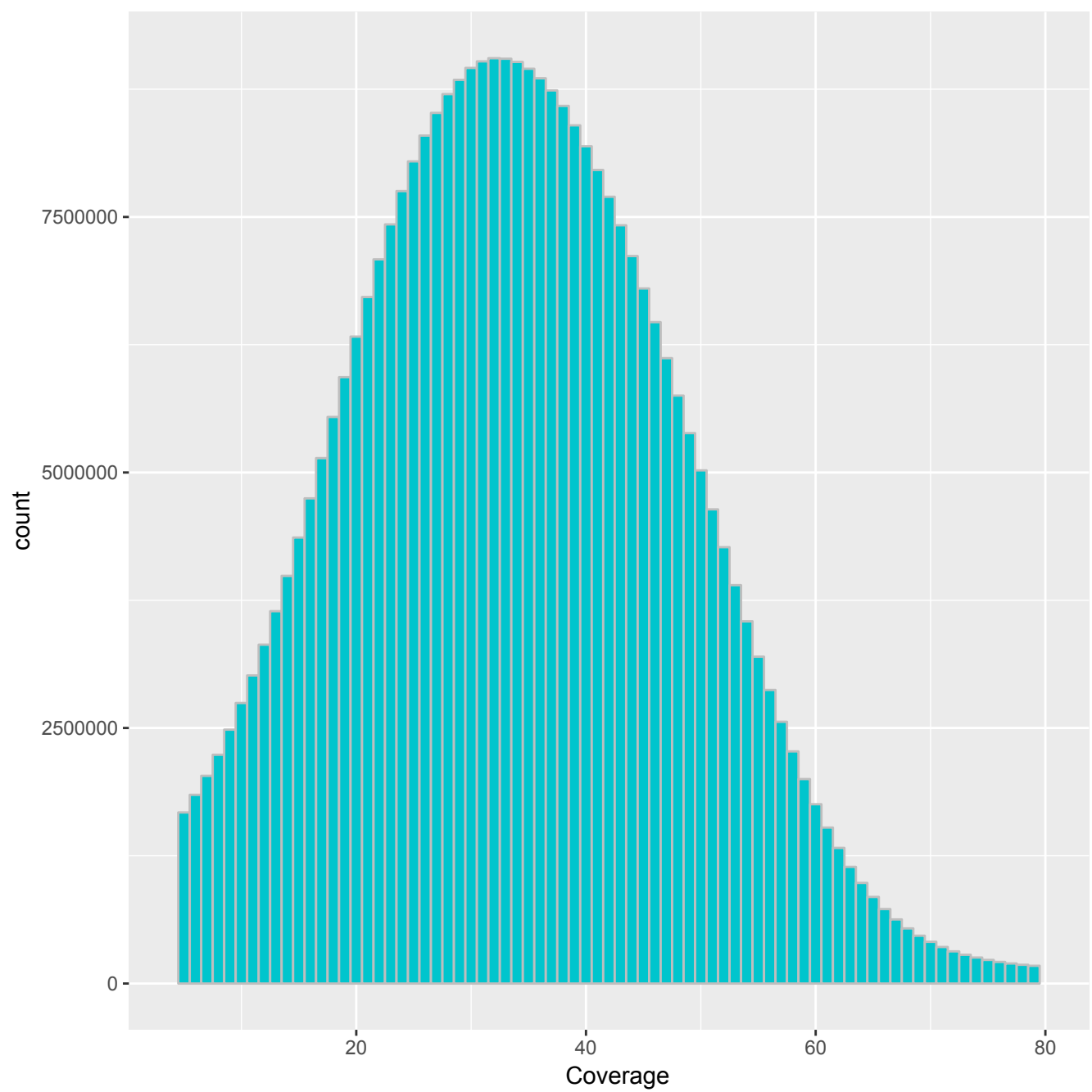

Supplement: Supplementary Figure 1 — Distribution of the coverage of each position in the assembly. The coverage of each position the S. mansoni genome assembly was calculated by Samtools, based on the filtered clean reads (see section Materials and Methods). [file Image1.PDF]

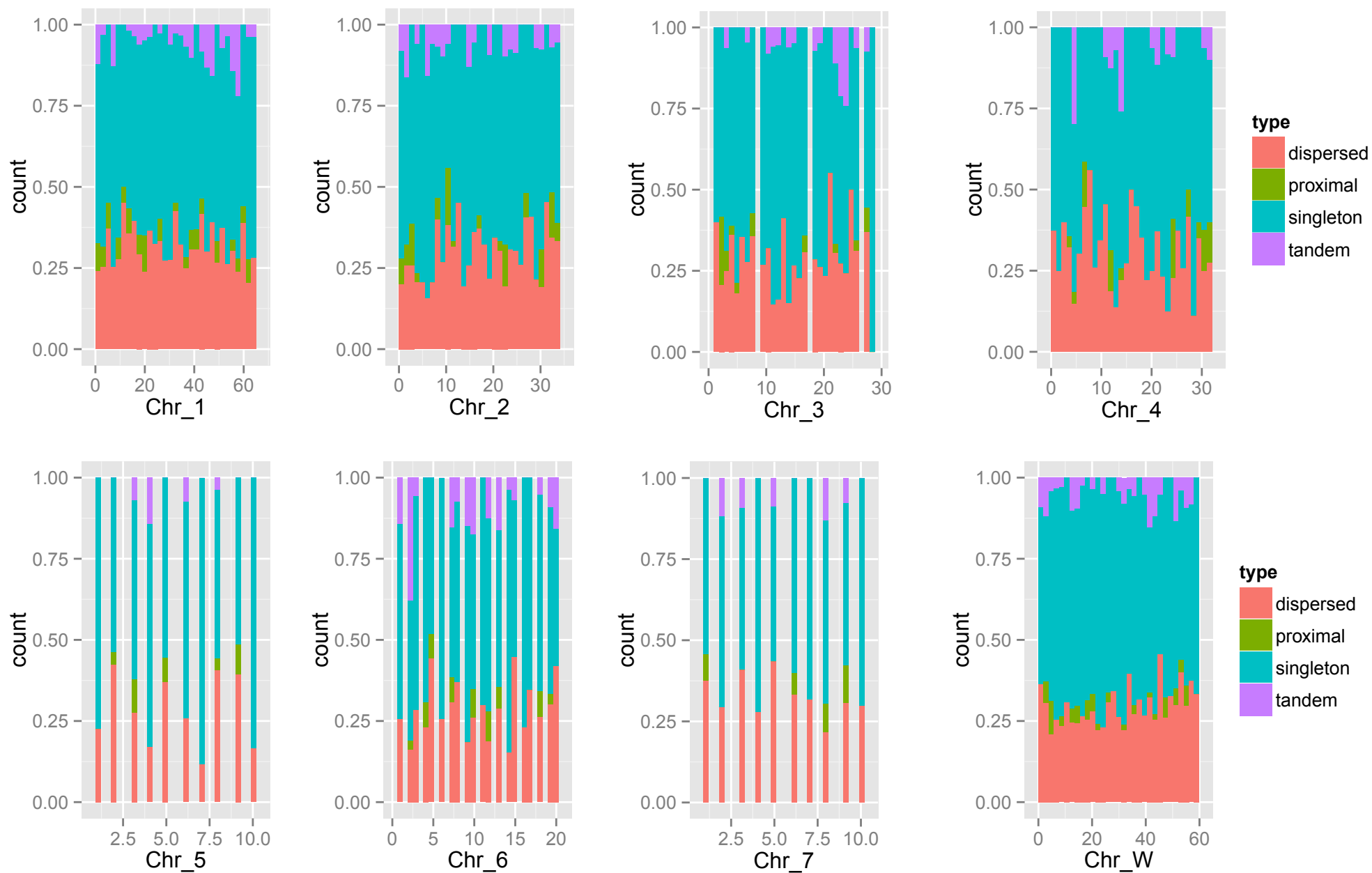

Supplement: Supplementary Figure 2 — Proportions of each duplication type along the chromosomes. The number of each duplication type (or singleton genes) within every continuous 1 Mb region along each chromosome was counted by a window-sliding analysis. The axis represents the locations at the end of each window (Mb). [file Image2.PDF]

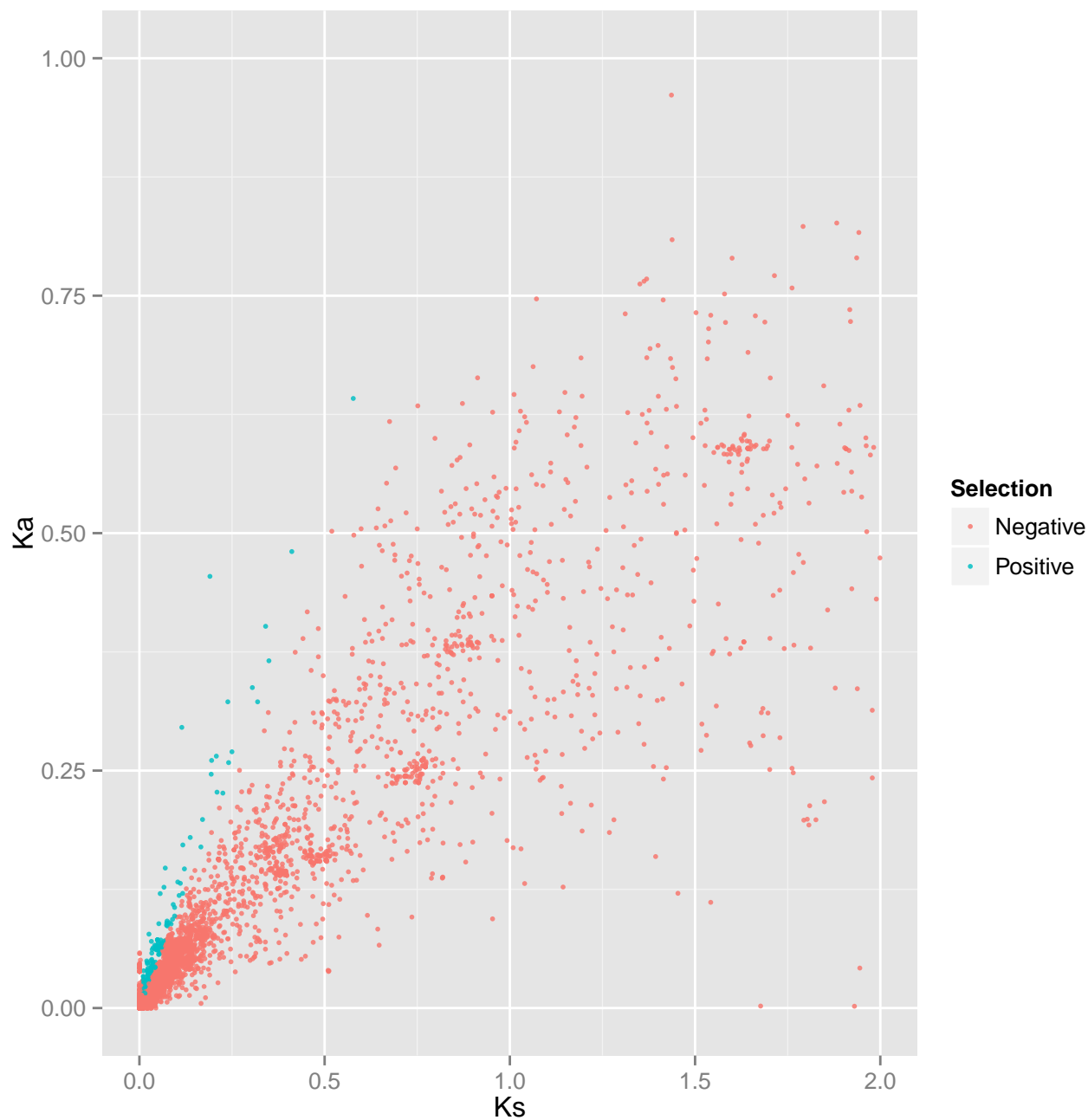

Supplement: Supplementary Figure 3 — Ka and Ks values for paralogous pairs. The dots with different colors represent genes under positive selection (blue) and under negative selection (red). [file Image3.PDF]
